# Supplementary material for: The Sensory and Motor Components of the Cortical Hierarchy Are Coupled to the Rhythm of the Stomach during Rest
Source: J Neurosci. 2022 Mar 16;42(11):2205–20. doi: 10.1523/JNEUROSCI.1285-21.2021 (PMC8936619; doi:10.1523/JNEUROSCI.1285-21.2021)
Supplement: Extended Data Figure 1-2 — Resting state networks according to the 7-network parcellation of Yeo et al (Yeo et al., 2011) and cortical regions synchronized with the stomach according to (Glasser et al., 2016) parcellation. Download Figure 1-2, DOCX file. [file ns-JN-RM-1285-21-s02.docx]

Figure 1-2 : Resting state networks according to the 7-network parcellation of Yeo et al (Yeo et al., 2011) and cortical regions synchronized with the stomach according to (Glasser et al., 2016) parcellation

| **RSN** | **Region name in atlas** | **Full name** | **OverlapLH** | **Effect sizes left** | **OverlapRH** | **Effect size right** |
| --- | --- | --- | --- | --- | --- | --- |
| Sensorimotor | 4 | Primary Motor Cortex | 47.8 | 0.82 | 47.7 | 0.78 |
| Sensorimotor | 3b | Primary Sensory Cortex | 50.4 | 0.74 | 52.9 | 0.79 |
| Sensorimotor | A1 | Primary Auditory Cortex | 61.8 | 0.67 | 64.8 | 0.61 |
| Sensorimotor | 5m | Area 5m | 62.0 | 0.53 | 7.0 | 0.46 |
| Sensorimotor | 5mv | Area 5 medio vental | 15.8 | 0.48 | 0.0 | - |
| Sensorimotor | 24dd | Dorsal Area 24d | 30.7 | 0.64 | 71.5 | 0.67 |
| Sensorimotor | 24dv | Ventral Area 24d | 5.8 | 0.37 | 25.7 | 0.54 |
| Sensorimotor | 1 | Area 1 | 53.2 | 0.77 | 38.2 | 0.75 |
| Sensorimotor | 2 | Area 2 | 22.6 | 0.65 | 1.0 | 0.39 |
| Sensorimotor | 3a | Area 3a | 45.5 | 0.70 | 57.6 | 0.77 |
| Sensorimotor | 6d | Dorsal area 6 | 59.4 | 0.75 | 62.7 | 0.71 |
| Sensorimotor | 6mp | Area 6mp | 27.9 | 0.56 | 24.8 | 0.59 |
| Sensorimotor | 6v | Ventral Area 6 | 9.9 | 0.49 | 15.1 | 0.45 |
| Sensorimotor | 43 | Area 43 | 79.0 | 0.68 | 37.8 | 0.61 |
| Sensorimotor | OP4 | Area OP4/PV | 64.2 | 0.61 | 35.3 | 0.50 |
| Sensorimotor | OP1 | Area OP1/SII | 73.6 | 0.61 | 25.9 | 0.56 |
| Sensorimotor | OP2-3 | Area OP2-3/VS | 29.3 | 0.55 | 41.2 | 0.54 |
| Sensorimotor | 52 | Area 52 | 25.6 | 0.52 | 64.2 | 0.62 |
| Sensorimotor | RI | RetroInsular Cortex | 80.4 | 0.65 | 24.9 | 0.54 |
| Sensorimotor | PFcm | Area PFcm | 57.6 | 0.58 | 40.8 | 0.51 |
| Sensorimotor | TA2 | Area TA2 | 0.8 | 0.40 | 47.8 | 0.66 |
| Sensorimotor | FOP2 | Frontal OPercular Area 2 | 1.3 | 0.34 | 29.8 | 0.54 |
| Sensorimotor | PBelt | ParaBelt Complex | 61.2 | 0.59 | 74.5 | 0.65 |
| Sensorimotor | A5 | Auditory 5 Complex | 19.0 | 0.58 | 36.5 | 0.79 |
| Sensorimotor | Ig | Insular Granular Complex | 42.3 | 0.57 | 83.0 | 0.63 |
| Sensorimotor | MBelt | Medial Belt Complex | 38.5 | 0.57 | 83.9 | 0.73 |
| Sensorimotor | LBelt | Lateral Belt Complex | 49.9 | 0.49 | 43.1 | 0.59 |
| Sensorimotor | A4 | Auditory 4 Complex | 20.7 | 0.50 | 47.3 | 0.63 |
| Visual | V1 | Primary Visual Cortex | 69.1 | 0.60 | 53.8 | 0.56 |
| Visual | V6 | Sixth Visual Area | 64.6 | 0.55 | 59.2 | 0.55 |
| Visual | V2 | Second Visual Area | 51.2 | 0.61 | 56.1 | 0.60 |
| Visual | V3 | Third Visual Area | 49.1 | 0.59 | 49.4 | 0.64 |
| Visual | V4 | Fourth Visual Area | 32.2 | 0.50 | 29.5 | 0.64 |
| Visual | V8 | Eighth Visual Area | 20.9 | 0.45 | 80.1 | 0.59 |
| Visual | V3A | Area V3A | 66.4 | 0.50 | 95.6 | 0.55 |
| Visual | V7 | Seventh Visual Area | 57.7 | 0.47 | 89.9 | 0.57 |
| Visual | FFC | Fusiform Face Complex | 0.0 | 0.25 | 23.0 | 0.72 |
| Visual | V3B | Area V3B | 80.1 | 0.47 | 13.5 | 0.43 |
| Visual | LO2 | Area Lateral Occipital 2 | 0.0 | - | 21.8 | 0.40 |
| Visual | PIT | Posterior InferoTemporal | 0.0 | - | 51.1 | 0.53 |
| Visual | MT | Middle Temporal Area | 0.0 | - | 26.7 | 0.48 |
| Visual | PreS | PreSubiculum | 0.0 | - | 6.9 | 0.42 |
| Visual | ProS | ProStriate Area | 69.0 | 0.60 | 44.9 | 0.54 |
| Visual | DVT | Dorsal Transitional Visual Area | 25.6 | 0.53 | 79.1 | 0.60 |
| Visual | V6A | Area V6A | 44.4 | 0.43 | 96.7 | 0.60 |
| Visual | VMV1 | VentroMedial Visual Area 1 | 30.0 | 0.46 | 50.0 | 0.57 |
| Visual | VMV3 | VentroMedial Visual Area 3 | 30.0 | 0.50 | 80.1 | 0.59 |
| Visual | V3CD | Area V3CD | 8.8 | 0.31 | 0.0 | - |
| Visual | LO3 | Area Lateral Occipital 3 | 0.0 | - | 17.0 | 0.38 |
| Visual | VMV2 | VentroMedial Visual Area 2 | 28.0 | 0.37 | 46.5 | 0.59 |
| Visual | VVC | Ventral Visual Complex | 25.2 | 0.67 | 31.9 | 0.64 |
| DMN | RSC | RetroSplenial Complex | 56.0 | 0.75 | 63.2 | 0.66 |
| DMN | SFROI | Superior Frontal Language Area | 26.2 | 0.50 | 24.7 | 0.51 |
| DMN | 7m | Area 7m | 0.0 | 0.30 | 13.0 | 0.46 |
| DMN | POS1 | Parieto-Occipital Sulcus Area 1 | 55.4 | 0.59 | 47.5 | 0.52 |
| DMN | 23d | Area 23d | 13.7 | 0.37 | 34.1 | 0.43 |
| DMN | v23ab | Area ventral 23 a+b | 55.1 | 0.51 | 37.4 | 0.53 |
| DMN | d23ab | Area dorsal 23 a+b | 38.6 | 0.47 | 51.9 | 0.49 |
| DMN | 31pv | Area 31p ventral | 0.6 | 0.24 | 8.0 | 0.35 |
| DMN | p32 | Area p32 | 0.0 | 0.33 | 7.7 | 0.44 |
| DMN | 10r | Area 10r | 17.8 | 0.44 | 11.5 | 0.47 |
| DMN | 8BROI | Area 8B Lateral | 0.0 | - | 16.7 | 0.53 |
| DMN | 10d | Area 10d | 21.2 | 0.57 | 36.8 | 0.62 |
| DMN | STGa | Area STGa | 0.0 | - | 12.0 | 0.57 |
| DMN | STSda | Area STSd anterior | 41.1 | 0.79 | 8.8 | 0.61 |
| DMN | STSdp | Area STSd posterior | 0.0 | - | 30.1 | 0.60 |
| DMN | STSvp | Area STSv posterior | 0.0 | - | 12.5 | 0.49 |
| DMN | TE1a | Area TE1 anterior | 4.9 | 0.32 | 0.3 | 0.34 |
| DMN | PGi | Area PGi | 0.0 | - | 7.4 | 0.42 |
| DMN | 31pd | Area 31pd | 0.0 | - | 15.9 | 0.42 |
| DMN | STSva | Area STSv anterior | 16.0 | 0.47 | 15.5 | 0.47 |
| DMN | TE1m | Area TE1 Middle | 0.0 | - | 23.5 | 0.54 |
| Saliency | 55b | Area 55b | 18.8 | 0.50 | 58.7 | 0.63 |
| Saliency | PSL | PeriSylvian Language Area | 0.5 | 0.33 | 6.6 | 0.49 |
| Saliency | STV | Superior Temporal Visual Area | 0.0 | - | 19.9 | 0.55 |
| Saliency | 5l | Area 5l | 7.4 | 0.52 | 20.0 | 0.55 |
| Saliency | 23c | Area 23c | 2.6 | 0.29 | 32.2 | 0.54 |
| Saliency | SCEF | Supplementary and Cingulate Eye Field | 53.8 | 0.70 | 73.3 | 0.68 |
| Saliency | 6ma | Area 6m anterior | 12.3 | 0.45 | 21.3 | 0.51 |
| Saliency | p24pr | Area Posterior 24 prime | 9.5 | 0.36 | 38.8 | 0.48 |
| Saliency | p32pr | Area p32 prime | 1.1 | 0.33 | 21.5 | 0.49 |
| Saliency | 6r | Rostral Area 6 | 0.5 | 0.56 | 22.9 | 0.57 |
| Saliency | PoI2 | Posterior Insular Area 2 | 0.5 | 0.41 | 36.4 | 0.67 |
| Saliency | FOP4 | Frontal OPercular Area 4 | 0.0 | - | 14.2 | 0.55 |
| Saliency | MI | Middle Insular Area | 0.0 | - | 21.1 | 0.50 |
| Saliency | FOP1 | Frontal OPercular Area 1 | 1.0 | 0.43 | 81.5 | 0.62 |
| Saliency | FOP3 | Frontal OPercular Area 3 | 0.0 | - | 11.8 | 0.37 |
| Saliency | TPOJ1 | Area TemporoParietoOcci pital Junction 1 | 0.0 | 0.32 | 7.0 | 0.52 |
| Saliency | PFop | Area PF opercular | 30.5 | 0.48 | 2.2 | 0.33 |
| Saliency | PoI1 | Area Posterior Insular 1 | 0.0 | - | 15.5 | 0.57 |
| Control | POS2 | Parieto-Occipital Sulcus Area 2 | 5.4 | 0.34 | 79.4 | 0.57 |
| Control | 7Pm | Medial Area 7P | 11.4 | 0.37 | 18.8 | 0.35 |
| Control | 8BM | Area 8BM | 2.0 | 0.20 | 10.4 | 0.49 |
| Control | 44 | Area 44 | 0.0 | - | 12.9 | 0.50 |
| Control | IFJa | Area IFJa | 0.0 | - | 23.4 | 0.42 |
| Control | IFSp | Area IFSp | 0.0 | - | 47.8 | 0.48 |
| Control | p9-46v | Area posterior 9-46v | 0.0 | - | 9.1 | 0.37 |
| Control | a9-46v | Area anterior 9-46v | 44.8 | 0.60 | 0.0 | - |
| Control | 9-46d | Area 9-46d | 6.7 | 0.53 | 0.0 | - |
| Control | a10p | Area anterior 10p | 43.1 | 0.74 | 0.0 | - |
| Control | 11ROI | Area 11l | 8.3 | 0.55 | 0.0 | - |
| Control | i6-8 | Inferior 6-8 Transitional Area | 2.6 | 0.25 | 9.5 | 0.40 |
| Control | s6-8 | Superior 6-8 Transitional Area | 0.0 | - | 23.0 | 0.53 |
| Control | TE1p | Area TE1 posterior | 0.0 | - | 10.4 | 0.47 |
| Control | 31a | Area 31a | 2.3 | 0.38 | 44.7 | 0.52 |
| Control | p10p | Area posterior 10p | 7.8 | 0.45 | 0.0 | - |
| Attention | FEF | Frontal Eye Fields | 29.8 | 0.60 | 52.7 | 0.62 |
| Attention | PEF | Premotor Eye Field | 0.0 | - | 11.0 | 0.44 |
| Attention | IPS1 | IntraParietal Sulcus Area 1 | 0.8 | 0.29 | 25.8 | 0.55 |
| Attention | PCV | PreCuneus Visual Area | 16.4 | 0.45 | 57.1 | 0.55 |
| Attention | 7Am | Medial Area 7A | 26.5 | 0.48 | 38.2 | 0.42 |
| Attention | 7PROI | Lateral Area 7P | 12.2 | 0.37 | 5.8 | 0.17 |
| Attention | 7PC | Area 7PC | 11.0 | 0.32 | 0.0 | - |
| Attention | VIP | Ventral IntraParietal Complex | 39.2 | 0.49 | 0.0 | - |
| Attention | 6a | Area 6 anterior | 13.3 | 0.52 | 0.3 | 0.46 |
| Attention | PFt | Area PFt | 1.7 | 0.34 | 3.8 | 0.29 |
| Attention | TPOJ3 | Area TemporoParietoOcci pital Junction 3 | 0.0 | - | 36.1 | 0.56 |
| Attention | PGp | Area PGp | 0.0 | - | 17.5 | 0.48 |
| Attention | IP0 | Area IntraParietal 0 | 12.2 | 0.34 | 2.4 | 0.42 |
| Limbic | 10v | Area 10v | 4.2 | 0.35 | 6.0 | 0.40 |
| Limbic | 10pp | Polar 10p | 30.2 | 0.60 | 0.0 | 0.37 |
